# Complement activation, placental malaria infection, and birth weight in areas characterized by unstable malaria transmission in central Sudan

**DOI:** 10.1186/s13000-015-0275-3

**Published:** 2015-05-06

**Authors:** Ammar Alim, Naser E.Bilal, Awad-Elkareem Abass, Elhassan M Elhassan, Ahmed A Mohmmed, Ishag Adam

**Affiliations:** Faculty of Medical laboratory Sciences, University of Khartoum, Khartoum, Sudan; Faculty of Medicine, University of Geziera, Medani, Sudan; Faculty of Medicine, Ribat University, Khartoum, Sudan; Faculty of Medicine, University of Khartoum, Khartoum, Sudan

**Keywords:** Malaria, Pregnancy, Complement, Hemoglobin, Birth weight, Sudan

## Abstract

**Background:**

The pathogenesis of malaria during pregnancy is not completely understood. There are few published data on complement activation and malaria during pregnancy. This study aimed to investigate complement activation and malaria during pregnancy, and their association with hemoglobin and birth weight.

**Methods:**

A cross-sectional study was conducted at Medani, Sudan. Soluble terminal complement complex (TCC) levels were measured using ELISA in maternal and cord blood samples from 126 parturient women.

**Results:**

There were no *Plasmodium falciparum*-positive blood films from maternal peripheral blood, the placenta, or cord blood samples. Three (2.4%) and 22 (17.5%) of the placentas showed chronic and previous infection with histopathological examination, respectively, while 101 (80.2%) of them had no malaria infection. The mean [SD] of the maternal (22.4 [6.1] vs. 26.5 [3.5] ng/ml, P < 0.001) and cord blood (24.5 [4.5] vs. 26.8 [4.4] ng/ml, P = 0.024) TCC levels were significantly lower in cases of placental malaria infection (n = 25) than in those without placental malaria infection (n = 101). Linear regression showed that placental malaria infection was significantly associated with birth weight (−0.353 g, P = 0.013), but there were no associations between maternal and cord TCC levels and maternal hemoglobin, or between TCC levels and birth weight.

**Conclusion:**

Maternal and cord blood TCC levels are lower in women with placental malaria infection than in those without placental malaria infection.

**Virtual Slide:**

The virtual slide(s) for this article can be found here: http://www.diagnosticpathology.diagnomx.eu/vs/9600054761463915

## Background

Malaria is a large public health problem in endemic tropical countries, especially sub-Saharan Africa. In sub-Saharan Africa, approximately 125 million pregnant women live in malaria-endemic areas and 32 million of these pregnant women are at risk of malaria [1,2]. Malaria during pregnancy can lead to adverse maternal and perinatal effects, mainly anemia and low birth weight (LBW) [3-5]. Pregnant Sudanese women are susceptible to malaria (even the severe form), regardless of their age and parity, and malaria is associated with anemia and LBW in these women [6-8].

The exact mechanism by which malaria infection and placental inflammation result in fetal growth restriction and LBW is poorly understood. However, many chemokines and inflammatory cytokines are associated with malaria infection and malaria-related LBW [9]. The complement system is an essential part of innate immunity and the host defense, and it plays a role in induction of inflammation and initiation of acquired immune responses in malaria-infected individuals [10,11]. C5a affects angiogenesis and its levels are increased with placental malaria infection [12-14]. Anaphylatoxin C5a and one C5b molecule I are produced when the terminal pathway is activated if C3b forms a complex with C3-convertases and generates C5-convertases, which can cleave C5. When C5b is associated with C6, C7, C8, and multiple C9 molecules, this leads to the generation of C5b-9, which is also known as the membrane attack complex. Interestingly, generation of C5b does not only lead to formation of membrane attack complex, but the major part is diverted by control proteins (e.g., clusterin and S protein) to form the C5b-9 complex, which is commonly referred to as the terminal complement complex (TCC) [15]. Recently, significantly higher levels of TCC were reported in women with *Plasmodium falciparum* placental malaria infection compared with the malaria-negative group [16]. Further investigation of the role of complement in the pathogenesis of malaria (and its effects) during pregnancy is necessary.

The current study was conducted in Medani Maternity Hospital, Central Sudan, to investigate TCC levels in women with placental malaria, and their effect on maternal hemoglobin and birth weight.

## Methods

A cross-sectional study was conducted during August to December 2011 (the rainy and post-rainy season) in the labor ward of the Medani Maternity Hospital. The area of this study is characterized by unstable malaria transmission. *P. falciparum* is the main malaria parasite species in the area and transmission occurs during the rainy (July to September) and post-rainy season [17]. Medani Maternity Hospital is a referral tertiary hospital caring for women who receive antenatal care at the hospital or are referred from other health centers and hospitals, and women who live close to the hospital facility. High-risk pregnancies are referred to the hospital. However, many women without a high-risk pregnancy deliver at this hospital.

The total sample size was calculated to have over 80% power to detect a difference of 5% at α = 0.05. We assumed that 10% of women might not respond or have incomplete data.

After obtaining signed informed consent from the patients, information on socio-demographics, history of obstetrics, medical history, antennal attendance characteristics, and bed net use was gathered using structured questionnaires. Body mass index was calculated by measuring maternal weight and height, which was expressed as weight (kg)/height (m)^2^. Newborns were weighed immediately following birth using the Salter scale and the sex of each newborn was recorded.

### Giemsa-stained blood smears for light microscopy

Maternal, placental, and cord blood films were prepared. Slides were stained by 10% Giemsa and the number of asexual parasites was counted per 200 leukocytes, assuming a leukocyte count of 8000 leukocytes/μl (for thick films) or per 1000 red blood cells (for thin films). Blood films were considered negative if no parasites were detected in 100 oil immersion fields of a thick blood film, which was double-checked in a blind manner by an expert microscopist. Maternal hemoglobin concentrations were estimated by the HemoCue hemoglobinometer (HemoCue AB, Angelhom, Sweden).

The blood (maternal and cord) was then allowed to clot and centrifuged for 10 minutes at 3000 rpm and the serum was separated and stored at - 20°C till the analyses.

### Placental histology

The details of how placental histology was performed have been mentioned previously [8,18,19]. Briefly, a 3-cm^3^ sample was obtained from the maternal surface approximately half the distance between the umbilical cord and the edge of the placenta. Each biopsy sample was immediately placed in 10% neutral buffered formalin. Buffer was used to prevent formation of formalin pigment, which has similar optical characteristics and polarized light activity as malaria pigment [20]. All of the biopsy samples were stored at room temperature until histology was performed. The placental biopsy samples were then processed and were embedded in paraffin wax, by standard techniques. In every case, paraffin sections that were 4-mm thick were stained with hematoxylin-eosin and Giemsa stains. Placental malaria infection was characterized using histology as previously described by Bulmer et al. as follows [21]: uninfected (no parasites or pigment), acute (parasites in intervillous spaces), chronic (parasites in maternal erythrocytes and pigment in fibrin, or cells within fibrin and/or chorionic villous syncytiotrophoblast or strom), and previous (no parasites, and pigment confined to fibrin or cells within fibrin). The slides were examined by a pathologist who remained blind regarding the clinical characteristics of these samples.

### ELISA for measuring TCC levels

Maternal and cord serum levels were measured using a human TCC ELISA kit (Biotain Pharma Co., Ltd., Xiamen City, Fujian Province, China) by following the manufacturer’s protocol.

### Statistical analysis

Data were entered into a computer using SPSS for windows (version 16.0). Continuous data (including TCC levels) were normally distributed and were compared between groups using Student’s *t* test. Multivariate analyses were performed using binary models for placental malaria infection as the dependent variable and linear models with hemoglobin, birth weight, and TCC (maternal and cord levels) levels as continuous dependent variables. Socio-demographic characteristics, education, antenatal care, residence, and placental malaria infections were the independent predictor of interest. Odds ratios (OR) and 95% confidence intervals (CI) were calculated and a P value of <0.05 was considered significant.

### Ethics

The study received ethical clearance from the Research Board at the Faculty of Medicine, University of Khartoum, Sudan.

## Results

Out of the 126 women enrolled in the study, 51 (41.5%) were primiparae and 62 (49.2%) had rural residency. Two (1.6%), 33 (26.2%), and 91 (92.1%) of these 126 women had no, one to two visits, and more than two visits of antenatal care, respectively. The majority (123; 97.6%) of them used bed nets during the index pregnancy.

Thirty one (24.6%) of these 126 women had blood group A, 17 (13.5%) had blood group B, three (2.4%) had blood group AB, and 75 (59.5%) had blood group O. The mean (SD) hemoglobin level was 10.7 (1.1) g/dl, and 72 (57.1%) of the women were anemic (hemoglobin <11 g/dl).

Eleven (8.7%) women delivered low-birth weight neonates (<2500 g).

### Malaria infections

There were no *P. falciparum*-positive blood films from maternal peripheral blood, the placenta, or cord blood samples. Three (2.4%) and 22 (17.5%) of the placentas showed chronic and previous infection on a histopathological examination, respectively, while 101 (80.2%) of them showed no signs of malaria infection. There were no significant associations between age, parity, residence, antenatal care, blood group, and placental malaria infection (Table 1).

**Table 1:** **Univariate and multivariate analyses of factors associated with placental malaria infection**

	**Univariate analysis**			**Multivariate analysis**		
**Variable**	**OR 95% CI P**			**OR 95% CI P**		
Age	0.9	0.9─1.0	0.152	1.0	0.9─1.1	0.524
Primiparae	0.8	0.7─1.0	0.185	1.2	0.3─4.0	0.714
Residence	0.6	0.3─1.4	0.696	1.1	0.3─3.7	0.785
Maternal education < secondary level	0.7	0.2─1.8	0.485	0.9	0.3─2.6	0.850
lack of antenatal care	1.3	0.6─2.5	0.242	1.0	0.3─3.4	0.920
Body mass index	1.0	0.9─1.0	0.668	1.1	0.8─1.3	0.377
Hemoglobin	0.3	0.1─0.8	0.016	0.7	0.4─1.1	0.201
Blood group O				0.9	0.3─2.5	0.941

Birth weight was significantly lower in women with placental malaria infection (n = 25) compared women who did not have placental malaria infection (n = 101; 2929.2 [648.9] vs 3206.6 [485.3] g, P = 0.023). In linear regression, placental malaria infection was significantly associated with birth weight (−0.353 g, P = 0.013, Table 2).

**Table 2:** **Linear regression analysis of factors associated with maternal hemoglobin and birth weight**

	**Maternal haemoglobin**			**birth weight**		
**Variable**	**Coefficient SE P**			**Coefficient SE P**		
Age	11.1	1.4	0.291	0.033	0.013	0.014
Parity	−0.114	0.104	0.275	−0.094	0.048	0.714
Residence	0.005	0.271	0.985	−0.013	0.126	0.920
Maternal education < secondary level	0.289	0.126	0.252	0.047	0.121	0.720
lack of antenatal care	0.266	0.272	0.330	−0.106	0.129	0.414
Body mass index	−0.067	0.047	0.158	0.010	0.022	0.668
Blood group O	−0.085	0.227	0.710	0.062	0.107	0.561
Placental malaria infection	−0.312	0.291	0.285	−0.353	0.139	0.013
Hemoglobin	─	─	─	0.051	0.044	0.247
Maternal terminal complement complex	0.006	0.027	0.839	0.022	0.013	0.091
Cord terminal complement complex	−0.025	0.026	0.338	−0.011	0.012	0.338

### Maternal and cord blood TCC levels

**Figure 1:**
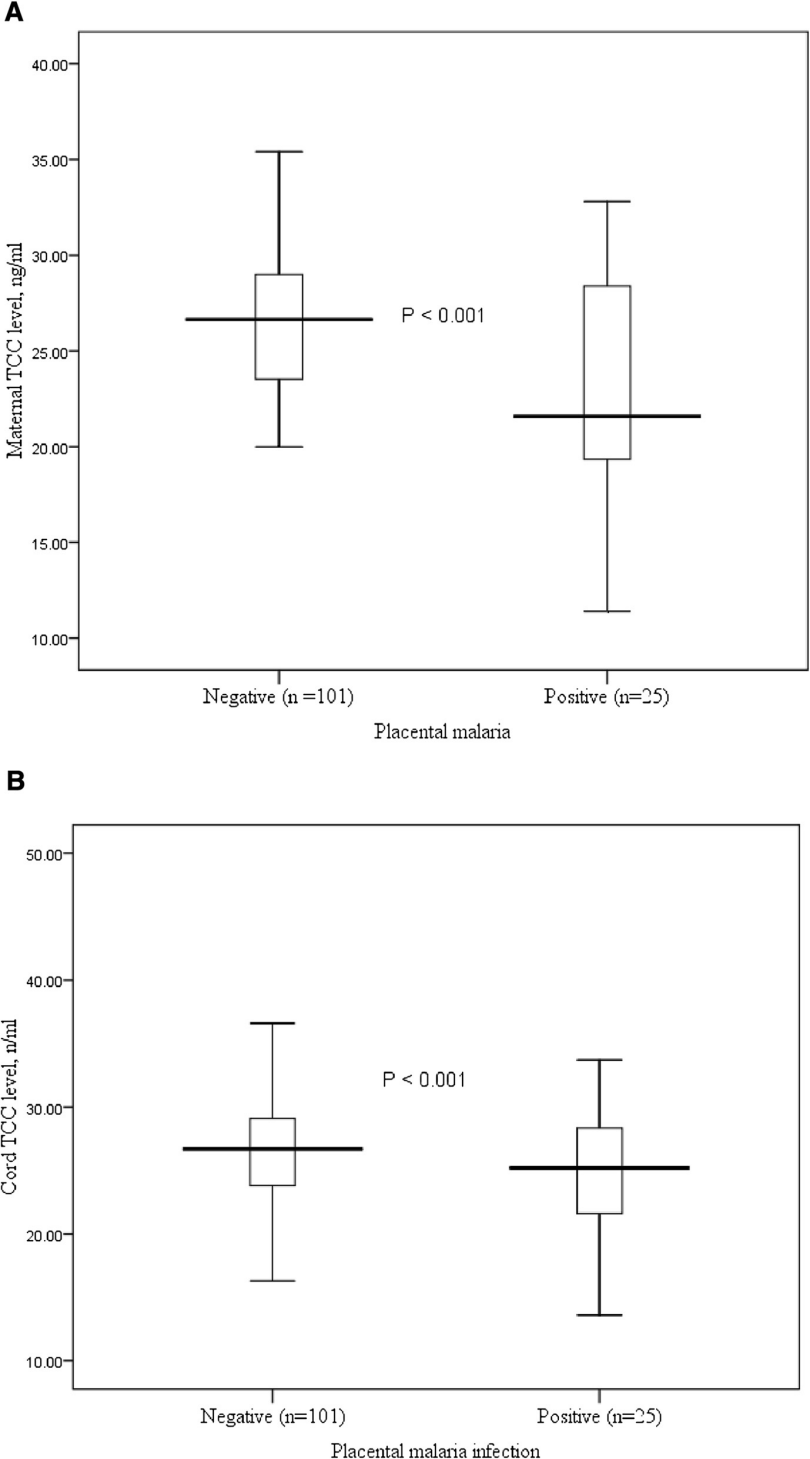
**Box plots of TCC levels in maternal and cord blood of women with placental malaria and in those without malaria in central Sudan.**

The mean [SD] of the maternal (22.4 [6.1] vs. 26.5 [3.5], P < 0.001) and cord (24.5 [4.5] vs. 26.8 [4.4], P = 0.024) blood TCC levels were significantly lower in women with placental malaria infection (n = 25) than in those without placental malaria infection (n = 101, Figure 1). Similarly, maternal (22.4 [6.1] vs. 26.5 [3.5], P < 0.001) and cord (24.5 [4.5] vs. 26.8 [4.4], P = 0.024) blood TCC levels were significantly lower in women who had blood group O than in those who had blood groups other than O.

In linear regression, placental malaria infection was associated with maternal (−4.091 mg, P < 0.001) and cord blood TCC levels (−0.353 mg, P = 0.013, Table 3).

**Table 3:** **Linear regression analysis of factors associated with TCC**

	**Maternal TCC**			**Cord TCC**		
**Variable**	**Coefficient SE P**			**Coefficient SE P**		
Age	0.147	0.084	0.084	0.033	0.013	0.014
Parity	0.623	0.936	0.507	−0.094	0.048	0.714
Body mass index	0.056	0.166	0.736	0.010	0.022	0.668
Blood group O	−1.839	0.773	0.019	0.062	0.107	0.561
Placental malaria infection	−4.091	0.958	<0.001	−0.353	0.139	0.013
Hemoglobin	−0.025	0.340	0.941	0.051	0.044	0.247
Maternal terminal complement complex	─	─	─	0.022	0.013	0.091

There was no significant difference between maternal and cord blood TCC levels (25.7 [4.4] vs 26.3 [4.5], P = 0.802) in infected and non-infected women, even when the malaria-infected women were analyzed separately (22.5 [6.3] vs. 24.7 [6.4], P = 0.138).

## Discussion

The main findings of the current study were that there were no associations between malaria placental infection and age or parity. Birth weight was significantly lower in newborns of malaria-infected women than in non-infected women. Furthermore, maternal and cord blood TCC levels were significantly lower in women with placental malaria infection than in those without placental malaria infection, and they had no effect on maternal hemoglobin levels and birth weight. The susceptibility of pregnant women to malaria infection, irrespective of their age or parity, is similar to previous findings in the same setting, as well as in different areas of Sudan [6,8,18,19,22,23].

In the current study, maternal and cord blood TCC levels were significantly lower in women with placental malaria infection than in those without placental malaria infection. Bayoumi *et al.* observed that cytokine levels (interferon-gamma, interleukin-4, and interleukin-10) were significantly lower in peripheral and placental sera of women with placental malaria infection in eastern Sudan [23]. Recently, high C5a levels were observed in placental malaria infection [13,14]. Likewise, Khattab *et al.* reported significantly higher levels of TCC in women with *P. falciparum* placental malaria infection compared with the malaria-negative group [16]. Notably, different types of malaria were investigated between studies, where previous placental malaria infection (using histology) was observed in the current study and microscopically-detected malaria was observed in the later study. Furthermore, differences in malaria endemicity should be taken onto account when comparing studies because malaria had unstable transmission in the current study and it was hyperendemic in the second setting.

Interestingly, in this study, maternal and cord blood TCC levels were significantly lower in women with an O blood group compared with those with other blood types. Although there was no association between the O blood group and placental malaria infection in the current study, a previous study in eastern Sudan showed that women with blood type O were at higher risk of placental malaria infection (OR = 1.9, 95% CI = 1.1–3.2) [19]. However, recently, the reverse finding was observed where there was a lower prevalence of placental malaria in primiparae with blood group O [24]. Recently, Rowe and colleagues observed that blood group O protects against severe *P. falciparum* malaria by the mechanism of reduced resetting [25].

In the current study, although placental malaria infection was associated with maternal and cord blood TCC levels and birth weight, there was no association between either maternal or cord blood TCC levels and birth weight. Recent observations have shown that TCC levels are higher in placental plasma samples of newborns weighing less than 2700 g than in heavier newborns [16]. Submicroscopic placental malaria (which was not investigated in the current study), rather than histology-detected malaria, might be the main determinant of LBW in this setting [8].

## Conclusion

There are significantly lower levels of maternal and cord blood TCC levels in women with placental malaria infection than in those without placental malaria infection. There is no association between maternal hemoglobin and TCC (maternal and cord blood levels) levels or between TCC levels and birth weight.
